# A functional difficulty and functional pain instrument for hip and knee osteoarthritis

**DOI:** 10.1186/ar2760

**Published:** 2009-07-09

**Authors:** Alan M Jette, Christine M McDonough, Pengsheng Ni, Stephen M Haley, Ronald K Hambleton, Sippy Olarsch, David J Hunter, Young-jo Kim, David T Felson

**Affiliations:** 1 Health & Disability Research Institute, Boston University School of Public Health, Boston University, 715 Albany Street – T5W, Boston, MA 02118, USA; 2 The Dartmouth Institute for Health Policy and Clinical Practice, Dartmouth College, 35 Centerra Parkway, Lebanon, NH 03766, USA; 3 Center for Educational Assessment, Department of Educational Policy, Research, and Administration, School of Education, University of Massachusetts, 813 North Pleasant Street, Amherst, MA 01003, USA; 4 Division of Research, New England Baptist Hospital, 125 Parker Hill Avenue, Boston, MA 02120, USA; 5 Harvard Medical School, Harvard University, 25 Shattuck Street, Boston, MA 02115, USA; 6 The Department of Orthopedic Surgery, Children's Hospital, 300 Longwood Avenue, Boston, MA 02115, USA; 7 Boston University School of Medicine, Boston University, 715 Albany Street R-304, Boston, MA 02118-2526, USA

## Abstract

**Introduction:**

The objectives of this study were to develop a functional outcome instrument for hip and knee osteoarthritis research (OA-FUNCTION-CAT) using item response theory (IRT) and computer adaptive test (CAT) methods and to assess its psychometric performance compared to the current standard in the field.

**Methods:**

We conducted an extensive literature review, focus groups, and cognitive testing to guide the construction of an item bank consisting of 125 functional activities commonly affected by hip and knee osteoarthritis. We recruited a convenience sample of 328 adults with confirmed hip and/or knee osteoarthritis. Subjects reported their degree of functional difficulty and functional pain in performing each activity in the item bank and completed the Western Ontario and McMaster Universities Osteoarthritis Index (WOMAC). Confirmatory factor analyses were conducted to assess scale uni-dimensionality, and IRT methods were used to calibrate the items and examine the fit of the data. We assessed the performance of OA-FUNCTION-CATs of different lengths relative to the full item bank and WOMAC using CAT simulation analyses.

**Results:**

Confirmatory factor analyses revealed distinct functional difficulty and functional pain domains. Descriptive statistics for scores from 5-, 10-, and 15-item CATs were similar to those for the full item bank. The 10-item OA-FUNCTION-CAT scales demonstrated a high degree of accuracy compared with the item bank (*r *= 0.96 and 0.89, respectively). Compared to the WOMAC, both scales covered a broader score range and demonstrated a higher degree of precision at the ceiling and reliability across the range of scores.

**Conclusions:**

The OA-FUNCTION-CAT provided superior reliability throughout the score range and improved breadth and precision at the ceiling compared with the WOMAC. Further research is needed to assess whether these improvements carry over into superior ability to measure change.

## Introduction

Patient functioning has become a standard measure of outcome in osteoarthritis (OA) care and research [1-3]. The wide range of function exhibited in this population makes it difficult to develop one functional outcome instrument for all patient groups [4,5]. Instruments with necessary measurement properties either are too extensive for clinical use or serve a limited patient population. An ideal measurement instrument would include the full range of functional activities relevant to OA treatment, with a sufficient number of response categories to measure meaningful change across the functional continuum [6].

Traditional administration methods require that a fixed set of questions be administered to all subjects. Under these conditions, trade-offs between comprehensive coverage and measurement precision with practicality for patients and clinicians have persistently challenged instrument developers and users. One potential solution to the need for high quality in measurement, comprehensive fixed-form instruments, has suffered from prohibitive respondent burden and administration costs [7,8]. Often, respondents must address redundant questions or those of low relevance [9-11]. The introduction of short-forms has raised concerns over decrements in score precision and over the ability of short-forms to measure clinically meaningful change [12]. Therefore, superior functional measures would improve the basis for valid judgments about the effectiveness of various OA treatments or for use in cohort studies.

Contemporary measurement theory and testing methods offer opportunities to overcome these significant challenges to measuring the effectiveness of clinical interventions. Item response theory (IRT) [13-15] provides a method for understanding the performance of specific questionnaire items across the range of function. This theory allows developers to design an extensive pool of superior questions covering all functional levels for each dimension.

The computer adaptive test (CAT) uses computer technology to minimize the number of questions required to find the functional level of the test-taker, allowing instrument developers to draw only the critical questions from a comprehensive pool of questionnaire items. The application of CAT methods combined with IRT methods was recently introduced in health measurement [6,16] and offers the potential to improve psychometric properties while reducing respondent burden and administrative costs. In CAT administration, after the subject responds to an initial item, the computer program selects and administers subsequent items based on the subject's previous responses, tailoring item selection to the subject. This iterative approach allows the selection of items that provide the most information at the respondent's current score estimate, thereby eliminating questions that are too hard or too easy. In this approach, all scores are on the same metric, regardless of the number of items administered, thus facilitating comparisons across time or across groups with different functional levels [17]. In this study, we developed a functional outcome instrument for hip and knee osteoarthritis research (OA-FUNCTION-CAT) using IRT and CAT methodologies and evaluated its psychometric performance compared with the full item bank and with the Western Ontario and McMaster Universities Osteoarthritis Index (WOMAC), the current standard in the field.

## Materials and methods

### Instruments

#### WOMAC

The WOMAC [18] is a 24-item questionnaire designed for use in lower extremity OA research. We used version LK3.0, with a 48-hour time frame and Likert scale. In this study, we did not use the two stiffness items from the WOMAC scale.

### OA-FUNCTION-CAT item bank development

#### Literature review

We performed a comprehensive literature review to yield functional activity instruments relevant to hip and knee OA, hand-searching the references provided in each paper to identify additional sources. We contacted the instrument developers to obtain the instruments and compiled the items as a resource for developing the preliminary item bank.

#### Patient focus groups

Experienced moderators conducted six semi-structured focus groups, each consisting of five or six patients with hip or knee OA, exploring patients' views on important outcomes for OA research. The sessions were audio-taped and transcripts were content-analyzed.

#### Clinician focus groups

We held three multi-disciplinary focus groups that included five or six clinicians with extensive expertise in the treatment of patients with OA.

#### Cognitive testing

The entire item bank was subjected to cognitive testing to discover problems with any items that would reduce instrument performance. Two groups of five or six adult patients with hip or knee OA were asked to read the instructions for clarity and assess a sample of the items for clarity and relevance. Cognitive testers asked standardized probe questions to identify difficulty in reading or comprehending instructions or items.

When focus group participants identified functional activities not covered by the item bank, new items were written. Further revisions were made based on cognitive testing results. The final item bank consisted of 125 functional activities commonly affected by hip or knee OA (Additional data file 1). The final rating scale asked the subject to report the amount of difficulty s/he had in doing each function as (a) none, (b) a little, or (c) a lot. Subjects also reported their pain severity in doing each activity as (a) none, (b) mild or moderate, or (c) severe. For those activities that a subject did not do, s/he reported whether (d) s/he did not do the activity because of the arthritis in her/his legs or (e) s/he did not do an activity for reasons other than the arthritis in her/his legs. The time frame was 'on an average day over the past month'. In previous work, we found that IRT models fit better when response categories are more distinct and item characteristic curves do not overlap or become disordered due to small frequencies of individual rating categories [19].

#### Study sample

We recruited a convenience sample of 328 adults from the greater Boston area with confirmed OA of the knee and/or hip from a pool of patients who had previously participated in OA research and from the practice of a local orthopedic surgeon. In all cases, confirmation of disease included evidence of OA on x-rays and frequent pain in the joint. For the knee joint, the x-ray protocol included either posteroanterior fluoroscopically positioned or metatarsophalangeal view (all semi-flexed) and they were read for the presence of a definite osteophyte. In most of the knees, lateral and/or skyline views were obtained to evaluate the patellofemoral joint. For the hip joint, the patient received a diagnosis of hip OA if any of the following were present: joint space narrowing, subchondral sclerosis, osteophytes, subchondral cyst, or symptomatic acetabular dysplasia.

#### Data collection

Eligibility was determined by telephone interview and included age of at least 18 years, English-speaking, pain or stiffness in the knee or hip within the prior month, radiographic evidence of a definite osteophyte for the knee or hip or joint space narrowing for the hip, or confirmation from the subject of a physician's diagnosis of knee or hip OA. Subjects were excluded if they had been diagnosed with rheumatoid arthritis, systemic lupus erythematosis, gout, or psoriatric arthritis or used a wheelchair to move about in their home. To ensure that we included a wide range of functional ability in the sample, we used the physical function scale of the short form-36 health survey (SF-36) to estimate and stratify subjects by functional level.

The OA-FUNCTION-CAT item bank and WOMAC were administered to the subjects in their homes by trained interviewers. We addressed potential order effects by counterbalancing the order of instrument administration. Demographic information (age, gender, ethnicity, race, education, and living and housing status) was collected for each subject, and gender-specific items were administrated to subjects of appropriate gender. For focus groups, cognitive testing, and the calibration study, informed consent was attained before participation, and all procedures were approved by the Institutional Review Board at Boston University.

#### OA-FUNCTION-CAT structure/domains

The underlying structure of functional pain and functional difficulty items was assessed using a series of confirmatory factor analyses [20]. We evaluated item loadings and residual correlations between items using MPlus software, version 3.12 [21]. We chose unweighted least squares (ULS) estimation based on polychoric correlation matrices and variance-adjusted estimation methods to improve the precision of our estimates given these skewed categorical data [20,22]. For each domain, we assessed eigenvalues associated with each factor extracted. Model fit was assessed using several approaches, including the chi-square test, comparative fit index (CFI), Tucker-Lewis index (TLI), and root mean square error approximation (RMSEA). For CFI and TLI, values range from 0 to 1, with higher values indicating better test model fit compared with a baseline model and with 0.90 or greater representing acceptable fit [23-25]. RMSEA represents misfit per degree of freedom (df), with lower values signifying better fit. Values of less than 0.05 suggest a 'very good fit', and values of around 0.08 are interpreted as 'marginal' fit. Values of greater than 0.1 are generally viewed as indicative of a 'poor fit' [26,27]. We examined the magnitude of the factor loadings on the primary factor and considered residual correlations; those of less than or equal to 0.20 (a) suggest that the primary factor explains the correlation between items and (b) indicate acceptable fit [28]. Higher correlations indicate violation of the local independence assumption.

#### Item calibrations

The generalized partial credit model (GPCM) was used to estimate the item calibrations for each domain [29-32]. We used weighted maximum likelihood (WML) estimation to estimate IRT-based scores for the functional pain and functional difficulty domains [22,33]. Item fit was evaluated using the likelihood ratio chi-square statistic (G^2^) for each item based on the comparison of expected and observed values across the distribution of the two domains. The likelihood ratio chi-square statistic for the whole test was examined to verify model fit of each domain, and Bonferroni-corrected *P *values were used in the significance tests. We standardized the scores estimated from the IRT model with a mean of 50 and standard deviation of 10. All of the IRT analyses were performed using the software package PARSCALE [34].

#### Differential item functioning

A fundamental assumption of IRT models is that a subject's score on an item should depend entirely on the subject's ability level in the relevant domain (for example, physical function) and the statistical characteristics of the item. Differential item functioning (DIF) means that, in spite of having the same underlying functional level, groups of subjects demonstrate different response probabilities, indicating that background variables (such as gender or site of OA) influenced the response [35]. A more severe pain on kneeling for subjects with knee OA compared with those with hip OA would be an example of DIF. We tested for the presence of DIF using logistic regression with background variables assigned as the independent variable and the OA-FUNCTION-CAT item score as the dependent variable. The analytic strategy successively added functional ability levels, background variables, and interaction terms to the model, and model comparison was based on the likelihood ratio test. The effect size of the DIF was classified based on the *R*^2 ^change between models [36]. Uniform DIF was identified when the background effect was significant but the interaction effect with the person's functional ability level was not, whereas non-uniform DIF was identified if the interaction effect was significant.

### Development of the simulated CAT program

Having finalized the item pool and generated item calibrations for each domain, we used HDRI™ software developed at Boston University to construct the OA-FUNCTION-CAT algorithms. The CATs were programmed to use WML score estimation and to select initial items in the middle of the ability ranges for pain and function. The program fed the response to the first item into the CAT algorithm and calculated a probable score and person-specific standard error (measure of precision). Subsequent items were selected and administered by the program until the preselected maximum number of items had been administered (in our analyses, 5-, 10-, or 15-item CATs were computer-selected). IRT assumes local independence of items, meaning that a subject's responses to any pair of items are statistically independent of each other [13]. One approach to local dependence is to remove items from the item bank. Rather than eliminating the items from the item bank, we used special programming within the CAT algorithm which allowed the selection of only one item within a set of locally dependent items.

### Psychometric evaluation of the OA-FUNCTION-CAT

To assess psychometric performance of the OA-FUNCTION-CAT, we conducted simulations to estimate scores for three fixed-length CATs (that is, 5, 10, and 15 items) and to compare their properties with those of the full item bank and the WOMAC. To make suitable comparisons between the WOMAC and the OA-FUNCTION-CATs and item banks, we first estimated calibrations for one instrument and then converted the other to the same scale, essentially calibrating the WOMAC items using the OA-FUNCTION-CAT item calibrations in the functional pain and functional difficulty domains as anchors. We compared mean scores generated by the CAT simulations with scores from the full item bank for the entire sample and by site of OA.

We considered the following characteristics in our analysis: accuracy, breadth of coverage, reliability, and precision. We assessed the accuracy of CATs relative to the full item bank by calculating Pearson correlation coefficients between each of the CAT-generated scores and the full item bank scores. To evaluate breadth of coverage, we calculated item distributions and percentage at the ceiling and floor for each scale of the full item bank compared with the WOMAC. We calculated expected values for each response category for each item and defined the range of the scale as the corresponding person's score estimates between the expected value of the lowest and highest response categories in each scale. Reliability represents the degree to which the differences across subject scores are due to real differences in pain or functional ability (true variance) as opposed to measurement error. At various positions on the scale, we examined the ratio of the true variance to the total variance for each instrument, using the following estimation: 1/1+(standard error)^2 ^[37]. Reliability was considered to be adequate for portions of the reliability function of greater than 0.70. Finally, precision was evaluated by calculating and comparing standard errors associated with each subject's score for the 5-, 10-, and 15-item CATs, the full item bank, and the WOMAC.

## Results

The average age of the study sample was 61.8 years (standard deviation 15.1), and 64.5% of participants were female (Table 1). The majority of patients had OA affecting only the knee (56.7%), a substantial minority reported both hip and knee involvement (27.1%), and the remaining 16.2% reported OA in the hip only.

**Table 1:** Characteristics of the study sample

Characteristics	Total sample n = 328
Mean age (standard deviation), years	61.8 (15.1)
Female	207 (64.5)
Race/Ethnicity	
American Indian or Alaskan native	1 (0.3)
Asian or Pacific Islander	8 (2.4)
Black (not of Hispanic origin)	30 (9.1)
Hispanic	4 (1.2)
Mixed race or ethnicity	2 (0.6)
Other or not available	4 (1.2)
White (not of Hispanic origin)	279 (85.0)
Education level	
≤ High school degree/GED	64 (19.5)
≤ 4 years of college	96 (29.3)
≥ 4 years of college	168 (51.2)
Joints affected	
Hip and knee	89 (27.1)
Hip only	53 (16.2)
Knee only	186 (56.7)
Joint surgery	
Yes	150 (45.7)
PF-10 score at baseline	
None to slightly limited	147 (44.8)
More limited	143 (43.6)
Most limited	38 (11.6)

Except for age, all values are presented as number (percentage) of subjects. GED, general equivalency diploma; PF, physical functioning.

### OA-FUNCTION-CAT domains

The results of confirmatory factor analyses using ULS supported the uni-dimensionality of each of the OA-FUNCTION-CAT functional domains (Additional data file 2). In the functional difficulty domain, a uni-dimensional model (chi-square (df) = 378 (102), *P *< 0.0001) provided the best fit across all 125 items, explaining 53% of the variance, and was easily interpretable. Only 3.4% of the residual covariances were greater than ± 0.20, which means that the local independence assumption was satisfied. The functional pain domain also fit a uni-dimensional model (chi-square (df) = 417 (104), *P *< 0.0001) and provided the best fit for the functional pain domain, explaining 55% of the variance. Only 3.06% of the residual covariances were greater than ± 0.2, satisfying requirements for local independence. The remaining fit statistics were very similar for the two domains: CFI values were 0.91 and 0.90 for functional difficulty and functional pain, respectively; TLI values were 0.96 for both; and RMSEA values were 0.090 and 0.096, respectively.

The data fit the GPCM, with the functional difficulty domain chi-square (df) = 1,342 (1,391), *P *= 0.83, and the functional pain domain chi-square (df) = 1,390 (1,343), *P *= 0.18. In terms of item fit, there was only one misfitting item (rising from a squatting position) in the functional difficulty pool and there were two misfitting items (washing the lower body and getting in and out of a tub) in the functional pain item pool.

### Differential item functioning

Two items displayed gender DIF in both the pain and difficulty domains (picking up a 25-pound weight from the floor and picking up a 25-pound weight from a table top). An additional item showed gender DIF in the functional difficulty domain (getting in and out of a truck, van, or sport utility vehicle [SUV]), and one other item demonstrated gender DIF in the functional pain domain (removing lower body clothing). Three items showed DIF by site of OA in both domains (getting into a kneeling position, getting out of a kneeling position, and shaving legs with a blade). Two additional items demonstrated DIF by site of OA in the functional difficulty domain (going down steps and rolling in bed), and seven other items exhibited DIF by site of OA in the functional pain domain (walking down three flights of stairs, getting into a squatting position, getting out of a squatting position, sitting on a bench for 20 minutes, going from sitting to lying down on a bed, moderate lifting, and washing and drying clothes). Only uniform DIF was detected in the analysis.

### Comparison of OA-FUNCTION-CAT with full item bank

**Figure 1:**
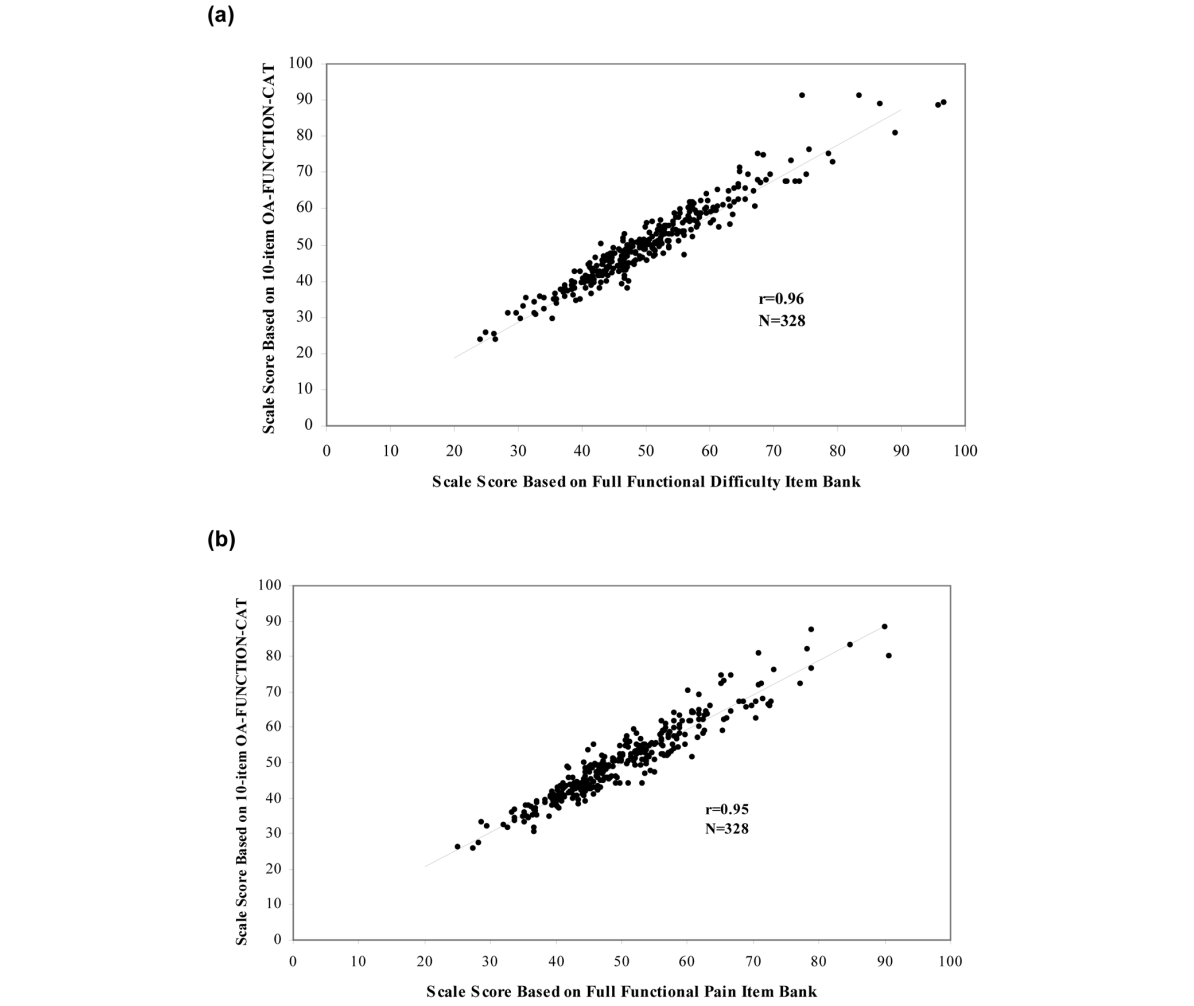
Correlation between the 10-item OA-FUNCTION-CAT scale scores with the full item bank. **(a) **Functional difficulty domain. **(b) **Functional pain domain. CAT, computer adaptive test; OA, osteoarthritis.

A high level of accuracy was observed in the Pearson correlation coefficients between the 5-, 10, and 15-item OA-FUNCTION-CATs and the full item banks, which were 0.92, 0.96, and 0.97, respectively, for functional difficulty, and 0.89, 0.95, and 0.97, respectively, for functional pain. Figure 1 provides score plots for the 10-item CAT and the full item bank. Descriptive statistics of scores from the 5-, 10-, and 15-item OA-FUNCTION-CATs were similar to those for the full item pool for both domains and for mean scores generated across OA conditions (Table 2). The standard errors of the 10-item CATs were consistently larger than the full item bank scores, reflecting the fewer number of items that were used to calculate the overall score.

**Table 2:** Comparison of scores from the 5-, 10-, and 15-item OA-FUNCTION-CATs and the full item bank

	Total (n = 328)		Both hip and knee (n = 89)		Hip (n = 53)		Knee (n = 186)	
				
Version	Mean (SD)	Range	Mean (SD)	Range	Mean (SD)	Range	Mean (SD)	Range
Difficulty								
5-CAT	49.7 (10.7)	23.7–82.3	47.2 (9.4)	31.6–79	52.3 (11.2)	35.8–82.3	50.1 (11)	23.9–82.3
10-CAT	50.1 (10.9)	23.7–91.2	47.6 (9.5)	31–88.6	53.4 (11.5)	37.9–91.2	50.4 (11.2)	23.7–91.2
15-CAT	50.4 (11.2)	22.7–95.6	47.8 (9.8)	30.2–91.8	53.8 (11.8)	37.4–95.6	50.6 (11.4)	22.7–95.6
Full item bank	50.4 (10.8)	24.2–96.7	47.6 (9.6)	28.5–95.8	54.1 (9.7)	37.5–75.7	50.7 (11.3)	24.2–96.7
Pain								
5-CAT	49.9 (10.7)	24.5–84.6	47.4 (8.9)	31.1–74.8	53.7 (11.9)	34.6–76.9	49.9 (10.8)	24.5–84.6
10-CAT	50.5 (11.5)	25.8–98.4	47.6 (8.6)	31.7–66.1	55.2 (13.1)	37.2–87.6	50.6 (11.8)	25.8–98.4
15-CAT	50.6 (11.8)	26.2–107.3	47.5 (8.6)	32.8–67.8	55.3 (13.3)	36.2–87.1	50.7 (12.3)	26.2–107.3
Full item bank	50.7 (11.9)	25–112.9	47.4 (8.2)	32.2–72.6	55.3 (12.5)	36.9–90.8	50.9 (12.7)	25–112.9

CAT, computer adaptive test; OA, osteoarthritis; SD, standard deviation.

### Comparison with the WOMAC

**Figure 2:**
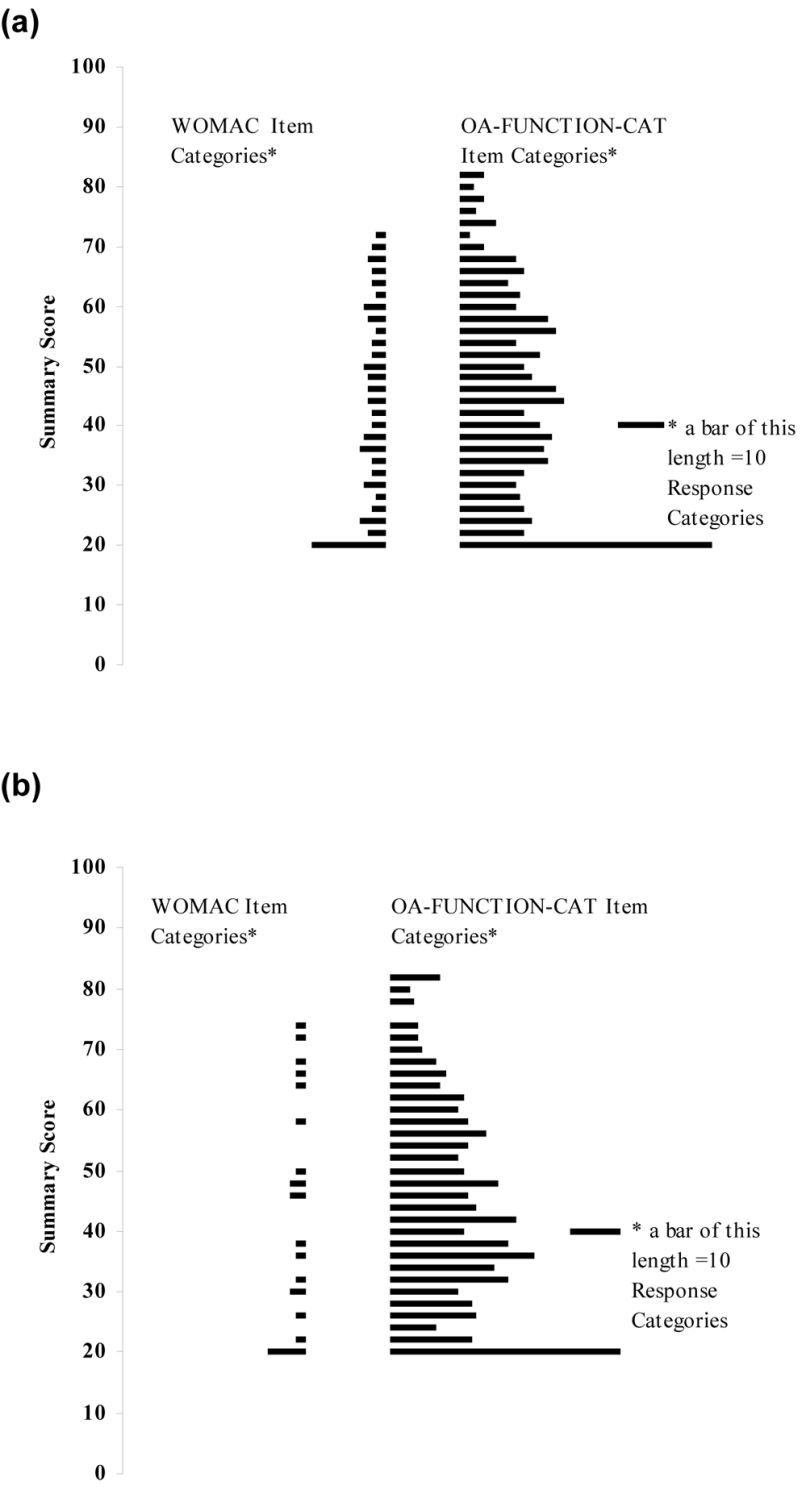
Comparison of the item distributions for the functional difficulty and functional pain scales of the OA-FUNCTION-CAT item bank with the WOMAC physical function and pain scales. Response categories are the expected values of each category within a rating scale, calculated for each item in the scale. In contrast to item thresholds, response category expected values are provided since they more accurately express the estimated range of the item across all of its categories. **(a) **WOMAC physical function scale versus OA-FUNCTION-CAT functional difficulty item bank. **(b) **WOMAC pain scale versus OA-FUNCTION-CAT functional pain item bank. CAT, computer adaptive test; OA, osteoarthritis; WOMAC, Western Ontario and McMaster Universities Osteoarthritis Index.

Breadth of item coverage for the OA-FUNCTION-CAT functional difficulty and functional pain item banks and corresponding WOMAC scales is shown in Figure 2. Item coverage is displayed as the range of a person's scores that correspond to the highest and lowest values of expected item response categories in each scale. Both OA-FUNCTION-CAT scales covered a broader estimated scoring range than did the WOMAC. For example, the highest item response category on the functional difficulty scale of the OA-FUNCTION-CAT was slightly over 80, whereas the WOMAC was limited to a little over 70 as its highest item response category expected score. Minimum item response category scores were similar for the two scales (approximately 20). Differences were more pronounced at the higher function/less pain end of the scales, where WOMAC lacked item coverage. The ceiling and floor calculations characterized this difference, particularly in the pain domain, where 21 (6.4%) of subjects were at the ceiling for the WOMAC pain scale compared with 2 (0.6%) for the OA-FUNCTION-CAT functional pain item bank. Similarly, there were 2 (0.6%) subjects at the ceiling for OA-FUNCTION-CAT functional difficulty compared with 10 (3%) for the WOMAC physical function scale. Neither scale displayed floor effects.

**Figure 3:**
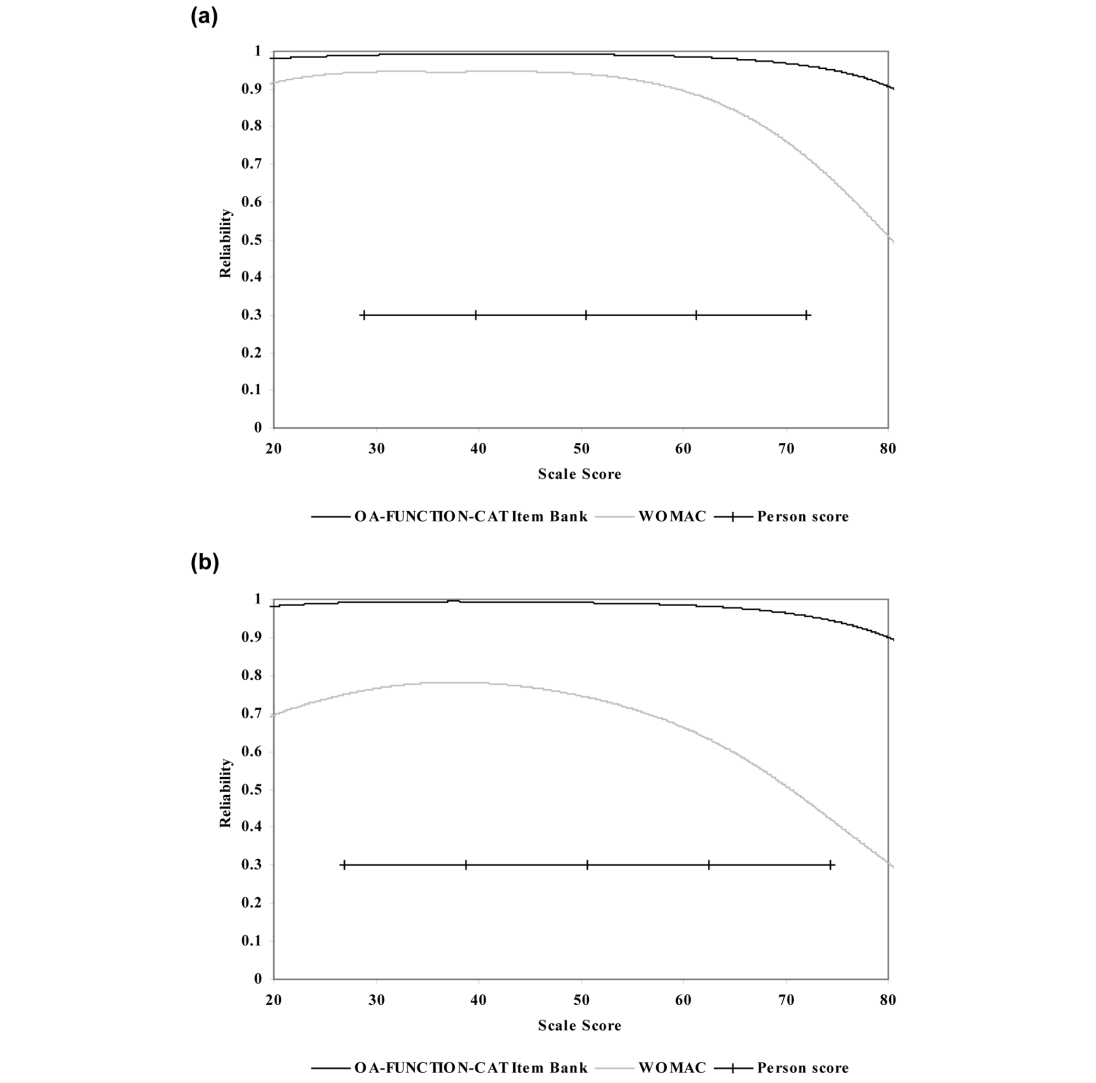
Comparison of the OA-FUNCTION-CAT item banks and WOMAC reliability estimates across the continuum of scale scores. **(a) **OA-FUNCTION-CAT functional difficulty item bank and WOMAC physical function scale. **(b) **OA-FUNCTION-CAT functional pain item bank and WOMAC pain scale. CAT, computer adaptive test; OA, osteoarthritis; WOMAC, Western Ontario and McMaster Universities Osteoarthritis Index.

The OA-FUNCTION-CAT item banks demonstrated very strong conditional reliability across the range of scores. For the functional difficulty scale, 95% of the sample scores achieved reliability estimates greater than 0.97 for the OA-FUNCTION-CAT compared with 0.76 for the WOMAC. For functional pain, 95% of OA-FUNCTION-CAT reliability estimates were over 0.96 versus 0.45 for the WOMAC pain scale (Figure 3).

**Figure 4:**
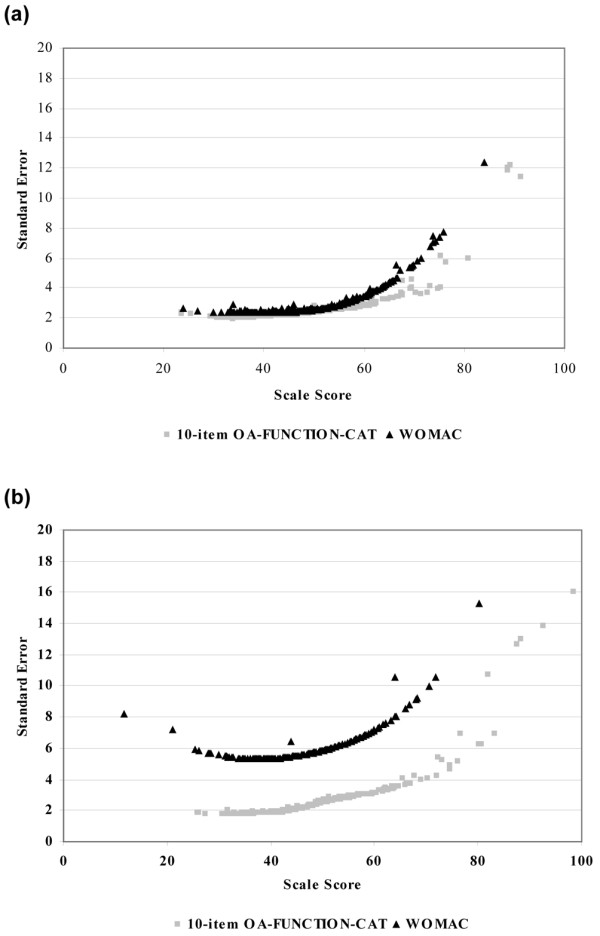
Precision of the 10-item OA-FUNCTION-CAT compared with the WOMAC scales as measured by standard errors of scale scores. **(a) **10-item OA-FUNCTION-CAT functional difficulty and WOMAC physical function scales. **(b) **10-item OA-FUNCTION-CAT functional pain and WOMAC pain scales. CAT, computer adaptive test; OA, osteoarthritis; WOMAC, Western Ontario and McMaster Universities Osteoarthritis Index.

The relative precision of the 10-item OA-FUNCTION-CAT and that of the WOMAC scales are shown in Figure 4 using the conditional standard error of measurement. For both domains, the OA-FUNCTION-CAT provided a higher degree of precision than the WOMAC across the range of scores. Superior performance of the OA-FUNCTION-CAT was more pronounced at the higher (better function/fewer symptoms) end of the scale and in the functional pain domain compared with functional difficulty.

## Discussion

This study of the OA-FUNCTION-CAT functional difficulty and functional pain item banks and CAT scales showed strong psychometric properties in this sample of persons with hip and/or knee OA. The full 125-item banks calibrated well with a uni-dimensional IRT model, providing greater breadth and more precise, more accurate, and more reliable estimates of functional difficulty and functional pain than the WOMAC. CAT performance remained close to that of the full item bank and superior to that of the WOMAC.

High correlations of the OA-FUNCTION-CAT item bank and the simulated CATs with the WOMAC can be viewed as one indication of the validity that the OA-FUNCTION-CAT item bank provides in characterizing the functional consequences of hip or knee OA. While the WOMAC has demonstrated acceptable measurement properties in this population, it was noted with a Rasch analysis that the items congregated in the center of the ability range, with several redundancies [38]. This is not surprising since focusing items in the mid range has been a common approach to the coverage/practicality dilemma within traditional survey construction methods. Our assessment of the breadth of the item banks indicated that the functional difficulty and functional pain item banks improved significantly on the content and scale coverage of the WOMAC.

Indeed, all measures of performance used in this study, including those for reliability and precision, showed improved function of the OA-FUNCTION-CAT over the WOMAC. More specifically, because of the focused effort to improve coverage, the greatest gains were achieved at the high end of the scales. Therefore, the OA-FUNCTION-CAT might be of particular benefit in capturing change among symptomatic patients at either end of the functional difficulty or functional pain domains. However, further improvements could be made to minimize the remaining ceiling effect noted in our analyses.

The results of these analyses are encouraging and consistent with prior studies indicating that the 10-item CATs have the ability to decrease time requirements for data collection requirements while enhancing psychometric properties [35,36]. However, these results are preliminary. Future research is needed to assess the administrative burden and the ability of OA-FUNCTION-CAT to detect smaller clinical and patient-relevant differences between groups and over time.

Our analyses with regard to DIF revealed some interesting results. Lifting heavy objects was more difficult for women than for men, and women reported greater levels of pain when lifting 25-pound objects than did men. Men reported more pain in getting clothes off, and women generally had more difficulty getting in and out of trucks or SUVs than did men. Subjects with knee OA had more difficulty than those with hip OA with items that involved stairs and squatting and kneeling. Those with both hip and knee OA had more difficulty with rolling and moving in bed and lower extremity self-care tasks than those who had only one joint affected (hips or knees). Adults with hip OA had more difficulty with moderate lifting than those with either knee or both joints affected. These predictable patterns of differences across different joint conditions suggest construct validity of our instrument.

The number of items that showed DIF by site of arthritis (hip versus knee) was relatively small for the functional domain but greater for the pain domain. These DIF findings revealed that the level of pain in certain activities (for example, climbing stairs, squatting, and kneeling) appears to be greater in the knee patients than in the hip patients. These results, if replicated in future research, may justify the development of separate calibrations for those items with DIF within different sites of OA. Given the limited sample size of patients in each type of OA in this sample, we did not feel justified in creating separate calibrations by site of arthritis. This is an issue that can be addressed in future research.

There are several alternatives for handling DIF. Removal of those items demonstrating DIF is one approach, leaving only those without DIF in the item bank. Unfortunately, this may eliminate items that contribute to the sensitivity and content validity of the resulting item banks. As an example, one alternative would be to develop separate sets of calibrations for hip and knee patients and for males and females and incorporate them into future CAT applications. This is an approach that we consider interesting for potential future research.

Several limitations of this research, including potential limits to the generalizability of this predominantly white, highly educated sample and a rather modest sample size for these analyses, should be acknowledged. In addition, different ethnic group ancestry was not examined. Given the level of CFI/RMSEA values, the structure of the OA-FUNCTION-CAT revealed in this study needs to be replicated in other samples with other sites of lower extremity OA. Similarly, a sample size of 328 subjects for these IRT analyses is acceptable if not ideal. One consequence of a relatively small sample size is that the person and item standard errors are larger than might be optimal for broader application of the item banks. Second, the effect of the relatively small number of unexpected responses for any particular item is more pronounced in a small sample, potentially leading to erroneously labeling an item as 'fitting'. For a two-parameter IRT model, it has been shown that a graded response model can be estimated based on 250 or more subjects [39]. From the item parameter recovery point of view, evidence suggests that increasing the number of items to be analyzed has little effect on the item parameter recovery but that increasing the number of categories will increase the error variance of the parameter estimates [40]. Given our relatively small number of categories (four), the sample size for these analyses is adequate.

Simulations of CAT scores, such as those used in this study, are possible whenever datasets include responses to all items in an item pool. Simulations are based on the assumption that the answers to a subset of those items selected using CAT would be identical to the answers given when embedded in a larger fixed-form instrument. Simulations are approximations of actual CAT administrations, and although they are likely to be good estimates, they may overestimate agreement between CAT and full item bank scores. Another area for future research is to assess the accuracy of CAT estimates in prospective studies.

## Conclusions

This study reveals that the functional difficulty and functional pain item banks provide superior reliability throughout the scale range and improved precision and coverage at the ceiling compared with a widely used functional measure in a sample of patients with hip or knee OA. Future work is needed to test the performance of the OA-FUNCTION-CAT prospectively and, in particular, to assess its responsiveness to clinically meaningful change. This preliminary study adds to the growing body of work pointing toward the CAT approach combined with IRT as a feasible solution to the persistent long-standing challenge of providing accuracy in outcome assessment and practicality of administration.

## Abbreviations

CAT: computer adaptive test; CFI: comparative fit index; df: degree of freedom; DIF: differential item functioning; GPCM: generalized partial credit model; IRT: item response theory; OA: osteoarthritis; RMSEA: root mean square error approximation; SUV: sport utility vehicle; TLI: Tucker-Lewis index; ULS: unweighted least squares; WML: weighted maximum likelihood; WOMAC: Western Ontario and McMaster Universities Osteoarthritis Index.

## Competing interests

SMH and AMJ hold stock interest in CRE Care LLC, (Gilford, NH USA), which distributes the OA-FUNCTION-CAT Instrument products. The other authors declare that they have no competing interests.

## Authors' contributions

AMJ conceived of and designed the study and participated in all aspects of the writing of the manuscript. CMM drafted the manuscript and coordinated the revisions. PN performed the statistical analysis and contributed to the writing of the manuscript, the response to reviewers, and the revisions. SMH contributed to the design of the study, the statistical analysis, the writing of the manuscript, and the response to reviewers. RKH contributed to the statistical analysis, the writing of the manuscript, and the revisions. SO supervised the data collection and contributed to the writing of the manuscript and the revisions. DH participated in the design and implementation of the study and the acquisition of data, contributed to the writing of the manuscript, and reviewed the revisions. Y-jK participated in the design and implementation of the study and the acquisition of data and reviewed the manuscript drafts and the revisions. DF participated in the design and implementation of the study, the acquisition of data, and the writing of the manuscript and reviewed the revisions. All authors read and approved the final manuscript.

## Supplementary Material

Additional file 1 A table listing the items in the OA-FUNCTION-CAT item bank and the item calibrations. Click here for file

Additional file 2 A figure of the scree plots for the OA-FUNCTION-CAT domains. Click here for file
