# Supplementary material for: A functional difficulty and functional pain instrument for hip and knee osteoarthritis
Source: Arthritis Res Ther. 2009 Jul 9;11(4):R107. doi: 10.1186/ar2760 (PMC2745788; doi:10.1186/ar2760)
Supplement: Additional file 1 — A table listing the items in the OA-FUNCTION-CAT item bank and the item calibrations. [file ar2760-S1.doc]

**OA–FUNCTION-CAT Items**

Item Listing:

In the Difficulty Scale the item stem is: “Because of the arthritis in your legs, how much difficulty did you have on an average day, over the past month when….?”

In the Pain Scale the item stem is: “Because of the arthritis in your legs, how much pain did you have, on an average day, over the past month when …..?”

| **No** | **Item** | **Factor Loading (Difficulty)*** | **Factor Loading (Pain)*** |
| --- | --- | --- | --- |
| 1 | Lying in bed. | 0.539 | 0.470 |
| 2 | Sitting down on a bed. | 0.610 | 0.660 |
| 3 | Standing up from a bed. | 0.655 | 0.617 |
| 4 | Turning in bed. | 0.534 | 0.532 |
| 5 | Sitting down on a toilet. | 0.638 | 0.685 |
| 6 | Getting up from a toilet. | 0.648 | 0.678 |
| 7 | Moving from sitting at the side of the bed to lying down on your back. | 0.724 | 0.694 |
| 8 | Rolling side to side in bed. | 0.558 | 0.482 |
| 9 | Moving from lying on your back to sitting on the side of the bed. | 0.669 | 0.635 |
| 10 | Climbing up one step with a railing. | 0.68 | 0.727 |
| 11 | Stepping into a stall shower. | 0.778 | 0.773 |
| 12 | Stepping out of a stall shower. | 0.788 | 0.766 |
| 13 | Climbing up 3-5 steps with a handrail. | 0.761 | 0.799 |
| 14 | Walking down 3-5 steps with a handrail. | 0.733 | 0.761 |
| 15 | Sitting down in a chair with arms (e.g. living room chair). | 0.657 | 0.758 |
| 16 | Standing up from a chair with arms (e.g. living room chair). | 0.693 | 0.769 |
| 17 | Standing for at least 1 minute. | 0.676 | 0.704 |
| 18 | Standing, while leaning on sink for 10 minutes. | 0.685 | 0.738 |
| 19 | Sitting on a bench without arms or back for 1 minute. | 0.677 | 0.726 |
| 20 | Sitting on a bench without arms or back for 20 minutes. | 0.658 | 0.645 |
| 21 | Sitting on a bench without arms or back for 1 hour. | 0.564 | 0.612 |
| 22 | Getting into a car or taxi. | 0.732 | 0.765 |
| 23 | Getting out of a car or taxi. | 0.693 | 0.686 |
| 24 | Stepping into a tub. | 0.773 | 0.732 |
| 25 | Stepping out of a tub. | 0.720 | 0.672 |
| 26 | Sitting down in an armless straight chair (e.g. dining room chair). | 0.696 | 0.733 |
| 27 | Standing up from an armless straight chair (e.g. dining room chair). | 0.696 | 0.714 |
| 28 | Getting into a truck, shuttle van, or sport utility vehicle. | 0.721 | 0.759 |
| 29 | Getting out of a truck, shuttle van, or sport utility vehicle. | 0.717 | 0.765 |
| 30 | Sitting down on a low, soft couch. | 0.648 | 0.706 |
| 31 | Standing up from a low, soft couch. | 0.660 | 0.693 |
| 32 | Walking one block. | 0.738 | 0.768 |
| 33 | Getting into a bus or truck that requires you to step up. | 0.784 | 0.789 |
| 34 | Getting out of a bus or truck that requires you to step down. | 0.782 | 0.761 |
| 35 | Climbing up a flight of stairs inside, using a handrail. | 0.748 | 0.805 |
| 36 | Walking down a flight of stairs inside, using a handrail. | 0.735 | 0.758 |
| 37 | Standing for 20 minutes. | 0.699 | 0.771 |
| 38 | Getting up from the floor (from a sitting or lying position), holding onto furniture. | 0.776 | 0.794 |
| 39 | Pulling open a heavy door. | 0.697 | 0.698 |
| 40 | Walking on an uneven surface (e.g. grass, dirt road, brick walkway, etc). | 0.791 | 0.823 |
| 41 | Walking 100 yards on a level surface. | 0.775 | 0.772 |
| 42 | Climbing up 3-5 steps without a handrail. | 0.752 | 0.821 |
| 43 | Walking down 3-5 steps without a handrail. | 0.723 | 0.793 |
| 44 | Climbing up 3 flights of stairs inside, using a handrail. | 0.79 | 0.757 |
| 45 | Walking down 3 flights of stairs inside, using a handrail. | 0.773 | 0.741 |
| 46 | Climbing up a flight of stairs outside, without a handrail. | 0.783 | 0.784 |
| 47 | Walking down a flight of stairs outside, without a handrail. | 0.740 | 0.742 |
| 48 | Getting into a squatting position (e.g. when gardening). | 0.646 | 0.680 |
| 49 | Getting out of a squatting position (e.g. when gardening). | 0.661 | 0.673 |
| 50 | Walking several blocks. | 0.792 | 0.823 |
| 51 | Getting into a kneeling position (e.g. when gardening). | 0.707 | 0.722 |
| 52 | Getting out of a kneeling position (e.g. when gardening). | 0.724 | 0.707 |
| 53 | Walking up a paved driveway or street. | 0.771 | 0.842 |
| 54 | Walking down a paved driveway or street. | 0.761 | 0.805 |
| 55 | Walking up a steep unpaved driveway. | 0.814 | 0.831 |
| 56 | Walking down a steep unpaved driveway. | 0.796 | 0.802 |
| 57 | Climbing stairs step-over-step without a handrail (alternating feet). | 0.771 | 0.787 |
| 58 | Bending over to pick something up off the floor. | 0.686 | 0.727 |
| 59 | Stooping to put something in a low cabinet. | 0.703 | 0.647 |
| 60 | Getting up from the floor without holding onto furniture. | 0.741 | 0.745 |
| 61 | Walking outdoors on a slippery surface. | 0.781 | 0.788 |
| 62 | Climbing a step ladder (4-5 steps). | 0.862 | 0.833 |
| 63 | Climbing several flights of stairs. | 0.812 | 0.805 |
| 64 | Sidestepping short distances (i.e. when working in the kitchen). | 0.696 | 0.693 |
| 65 | Hiking a mile on uneven terrain. | 0.785 | 0.748 |
| 66 | Sitting in a car for 10 minutes. | 0.641 | 0.745 |
| 67 | Sitting in a car for 30 minutes. | 0.63 | 0.685 |
| 68 | Sitting in a car for 1 hour. | 0.528 | 0.645 |
| 69 | Sitting in a car for more than 1 hour. | 0.567 | 0.608 |
| 70 | Walking a mile or more. | 0.767 | 0.774 |
| 71 | Walking a mile at a brisk pace without stopping to rest. | 0.791 | 0.769 |
| 72 | Walking 3 or more miles. | 0.775 | 0.732 |
| 73 | Running 1 mile. | 0.722 | 0.571 |
| 74 | Running 5 or more miles. | 0.675 | 0.488 |
| 75 | Making sharp turns when running. | 0.705 | 0.646 |
| 76 | Starting quickly with running. | 0.682 | 0.583 |
| 77 | Changing directions quickly (cutting, twisting, pivoting) while running. | 0.736 | 0.667 |
| 78 | Coming to a quick stop when running. | 0.708 | 0.654 |
| 79 | Hopping on your right leg. | 0.638 | 0.623 |
| 80 | Jumping/landing on your right leg. | 0.677 | 0.611 |
| 81 | Shaving your legs with a blade razor. | 0.542 | 0.613 |
| 82 | Putting on pantyhose. | 0.658 | 0.661 |
| 83 | Putting on long pants. | 0.735 | 0.806 |
| 84 | Taking off long pants. | 0.718 | 0.721 |
| 85 | Washing your legs when taking a bath. | 0.761 | 0.759 |
| 86 | Washing your legs when taking a shower. | 0.753 | 0.796 |
| 87 | Drying your legs. | 0.755 | 0.807 |
| 88 | Putting on socks. | 0.647 | 0.667 |
| 89 | Putting dishes away in a cupboard. | 0.789 | 0.791 |
| 90 | Operating a washer, including loading and unloading clothes. | 0.781 | 0.745 |
| 91 | Tying shoes. | 0.733 | 0.728 |
| 92 | Cutting your toenails. | 0.682 | 0.730 |
| 93 | Moving up in bed (e.g. reposition self). | 0.650 | 0.650 |
| 94 | Walking around in your bathroom. | 0.859 | 0.858 |
| 95 | Walking around one floor of your home. | 0.796 | 0.853 |
| 96 | Walking around one floor of a doctor’s office building. | 0.809 | 0.833 |
| 97 | Picking up clothes from the floor, while standing, without holding onto anything. | 0.757 | 0.752 |
| 98 | Walking quickly indoors to answer a telephone. | 0.758 | 0.743 |
| 99 | Walking through an airport or mall carrying something. | 0.789 | 0.782 |
| 100 | Walking across an intersection of a street with timed lights. | 0.772 | 0.818 |
| 101 | Opening a high window above shoulder height while standing. | 0.745 | 0.719 |
| 102 | Carrying a chair from one room to another. | 0.806 | 0.827 |
| 103 | Loading packages in a car trunk or hatchback. | 0.818 | 0.815 |
| 104 | Unloading packages from a car trunk or hatchback. | 0.826 | 0.842 |
| 105 | Carrying a fairly heavy item while walking. | 0.782 | 0.841 |
| 106 | Carrying a large object while walking. | 0.797 | 0.808 |
| 107 | Carrying an unsteady object, requiring two hands (e.g. a tray of food) while walking. | 0.733 | 0.782 |
| 108 | Lifting a suitcase by the handle, walking from house to car. | 0.791 | 0.819 |
| 109 | Lifting 25 pounds (e.g. dog food or a large bag of fertilizer) from the floor. | 0.731 | 0.731 |
| 110 | Lifting 25 pounds (e.g. dog food or a large bag of fertilizer) from a table. | 0.745 | 0.761 |
| 111 | Carrying groceries. | 0.832 | 0.825 |
| 112 | Carrying a large object like a laundry basket in both arms while climbing a flight of stairs. | 0.793 | 0.773 |
| 113 | Stepping up a curb. | 0.796 | 0.804 |
| 114 | Stepping down a curb. | 0.751 | 0.781 |
| 115 | Stepping over a one foot high object (e.g. a low fence). | 0.815 | 0.807 |
| 116 | Working the car pedals while driving. | 0.557 | 0.649 |
| 117 | Cleaning up spills on the floor with a rag. | 0.752 | 0.759 |
| 118 | Cleaning up spills on the floor with a mop. | 0.762 | 0.830 |
| 119 | Sweeping the floor. | 0.816 | 0.809 |
| 120 | Cleaning the floor using a broom and dustpan. | 0.764 | 0.780 |
| 121 | Making a bed, including spreading and tucking bed sheets, while standing. | 0.774 | 0.801 |
| 122 | Carrying 2 plastic grocery bags with handles at your side for 20 yards. | 0.78 | 0.793 |
| 123 | Crossing the road at a 4-lane traffic light with curbs. | 0.810 | 0.815 |
| 124 | Sitting down in a tub | 0.758 | 0.723 |
| 125 | Taking off socks | 0.653 | 0.661 |

*: Standardized Factor Loading
