# Supplementary material for: A functional difficulty and functional pain instrument for hip and knee osteoarthritis
Source: Arthritis Res Ther. 2009 Jul 9;11(4):R107. doi: 10.1186/ar2760 (PMC2745788; doi:10.1186/ar2760)
Supplement: Additional file 2 — A figure of the scree plots for the OA-FUNCTION-CAT domains. [file ar2760-S2.doc]

**Scree plots with eigenvalues for each domain of the OA-FUNCTION-CAT.**
